# Unusual presentation of spontaneous pneumomediastinum: a case report

**DOI:** 10.1186/1757-1626-1-349

**Published:** 2008-11-24

**Authors:** Sheharyar Asif Qureshi, Andrew Tilyard

**Affiliations:** 1 Specialty Registrar, Emergency Admissions Unit, Lincoln County Hospital, Lincoln, UK; 2 Consultant Intensive Care Unit, Derriford Hospital, Plymouth NHS Trust, South Devon, UK

## Abstract

The diagnosis of spontaneous pneumomediastinum in an acute hospital setting can present as a challenge. We present a case of 32 year Caucasian male with gradual swelling of his face and neck with increasing hoarseness of voice. He was treated for anaphylaxis with little improvement. He underwent a video-assisted thoracoscopic surgery procedure (VATS) with a definite diagnosis of sub pleural bleb.

## Introduction

Spontaneous pneumomediastinum is an uncommon benign condition, which should be diagnosed with the high degree of suspicion [1].

## Case presentation

We present a 34 year old Caucasian male, who worked as a plumber. He was admitted for the third time in four weeks with gradual worsening of the swelling of his left eye, face and neck. He suffered with increasing hoarseness of voice on this occasion. He had no significant past medical and family history. He was a non smoker and drank alcohol socially. He was taking no regular medications.

He was treated on these presentations for anaphylaxis with little improvement. His earlier serial chest X rays and CT scan of the chest were uneventful. He had an uneventful recovery on previous occasions and eventually discharged home.

**Figure 1:**
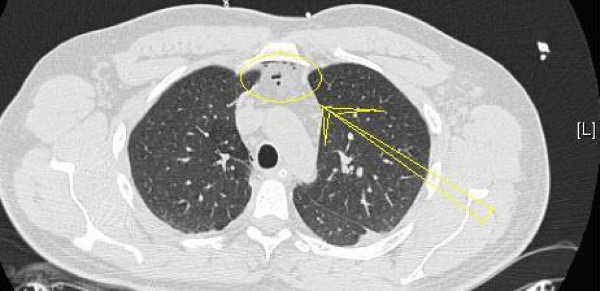
CT thorax of this patient demonstrating pneumomediastinum.

On this occasion he was unable to open his eyes but had palpable surgical emphysema on the face, neck and chest. His physical examination revealed a clear chest. His routine haematology and biochemistry results were normal. His repeat CT thorax revealed pneumomediatinum (Figure 1). His barium swallow and fiberoptic bronchoscopy revealed no abnormalities. The culture of bronchoalveolar-lavage was uneventful.

He underwent a video-assisted thoracoscopic surgery (VATS) procedure with a definite diagnosis of sub pleural bleb. There were no further untoward complications.

## Conclusion

Spontaneous pneumomediastinum presents with non specific signs and symptoms but certain modern and less invasive techniques can be used to aid early diagnosis [2]. Such advice would inform and support those specialists involved in managing this potentially serious condition.

## Competing interests

The authors declare that they have no competing interests.

## Authors' contributions

SQ collected the data and drafted the manuscript. AT provided the supervision for writing this manuscript. All authors read and approved the manuscript.

## Consent

Written informed consent was obtained from the patient for publication of this case report and accompanying images. A copy of the written consent is available for review by the Editor-in-Chief of this journal
